# Supplementary material for: Predicting Risk of Antenatal Depression and Anxiety Using Multi-Layer Perceptrons and Support Vector Machines
Source: J Pers Med. 2021 Mar 12;11(3):199. doi: 10.3390/jpm11030199 (PMC8000443; doi:10.3390/jpm11030199)
Supplement: Supplementary file 1 [file jpm-11-00199-s001.pdf]

Supplementary Materials

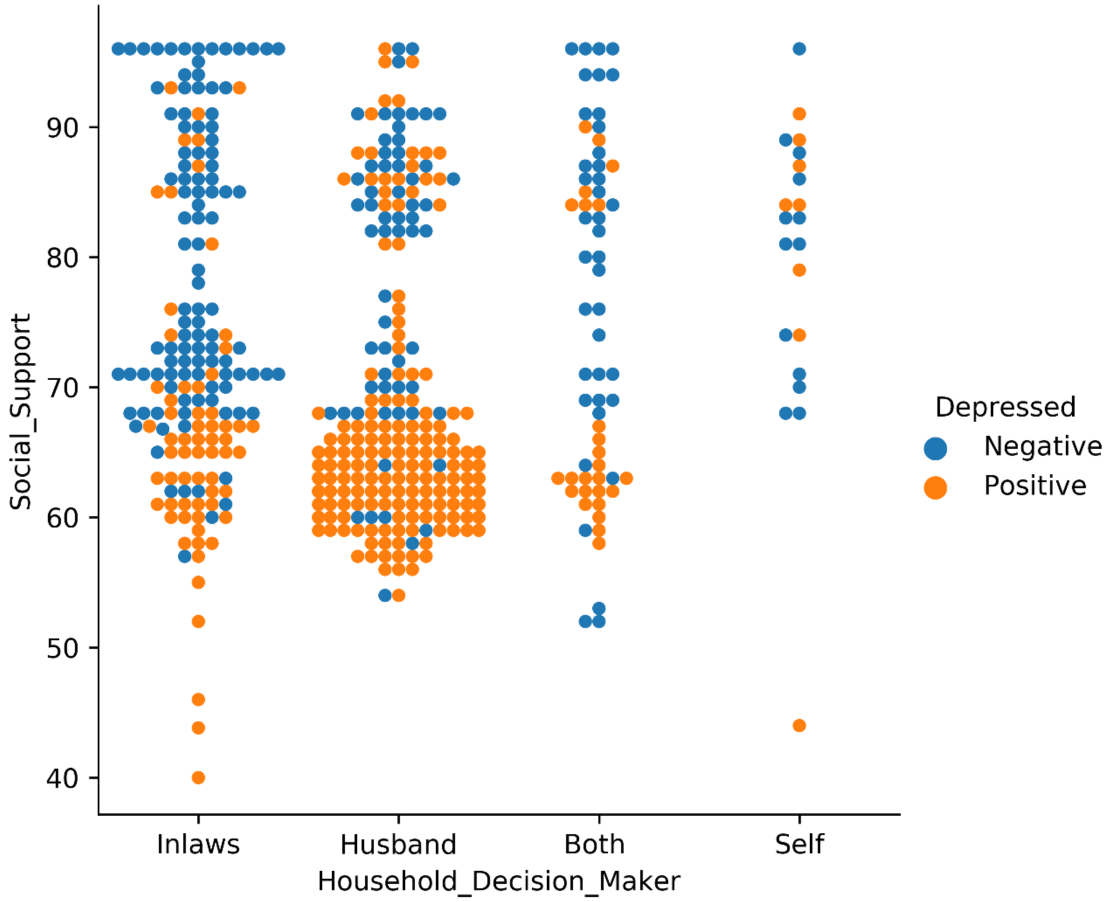

Figure S1 Association between social support, decision making and status of depression.

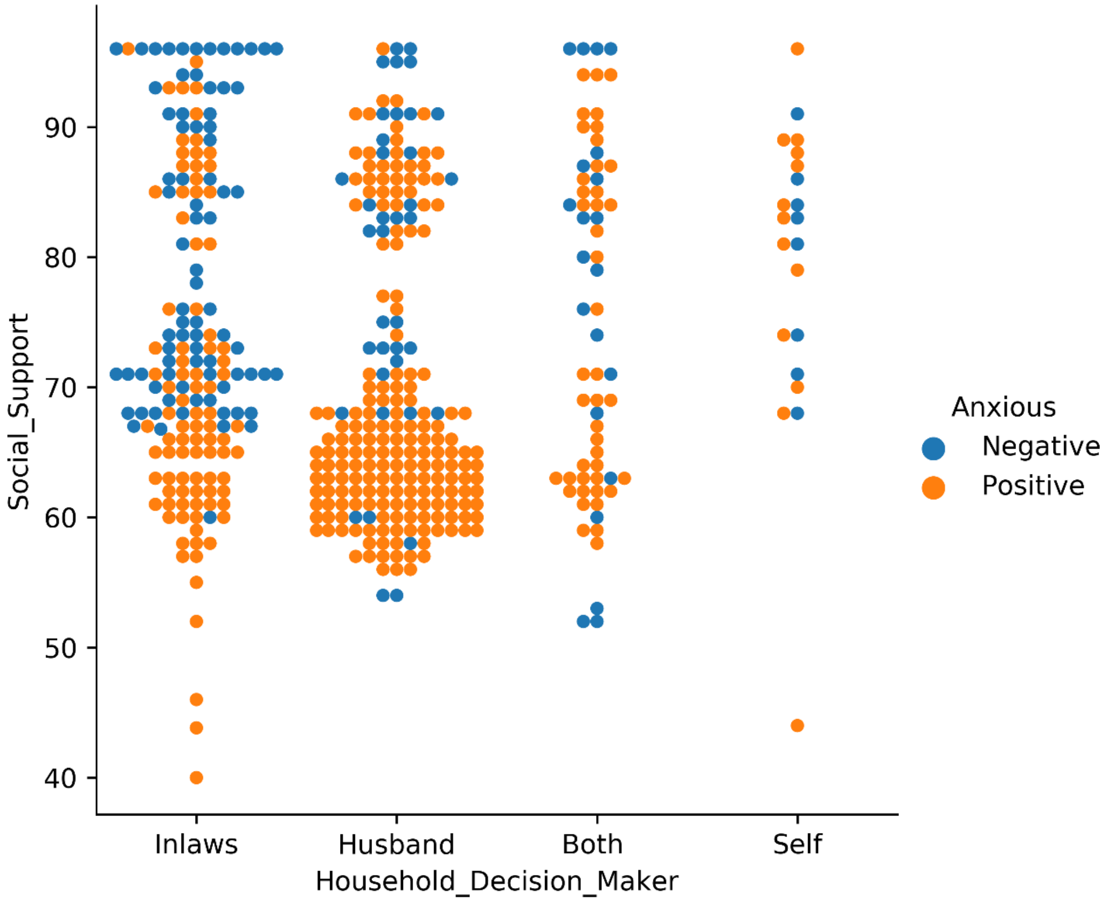

Figure S2 Association between social support, decision making and status of anxiety.

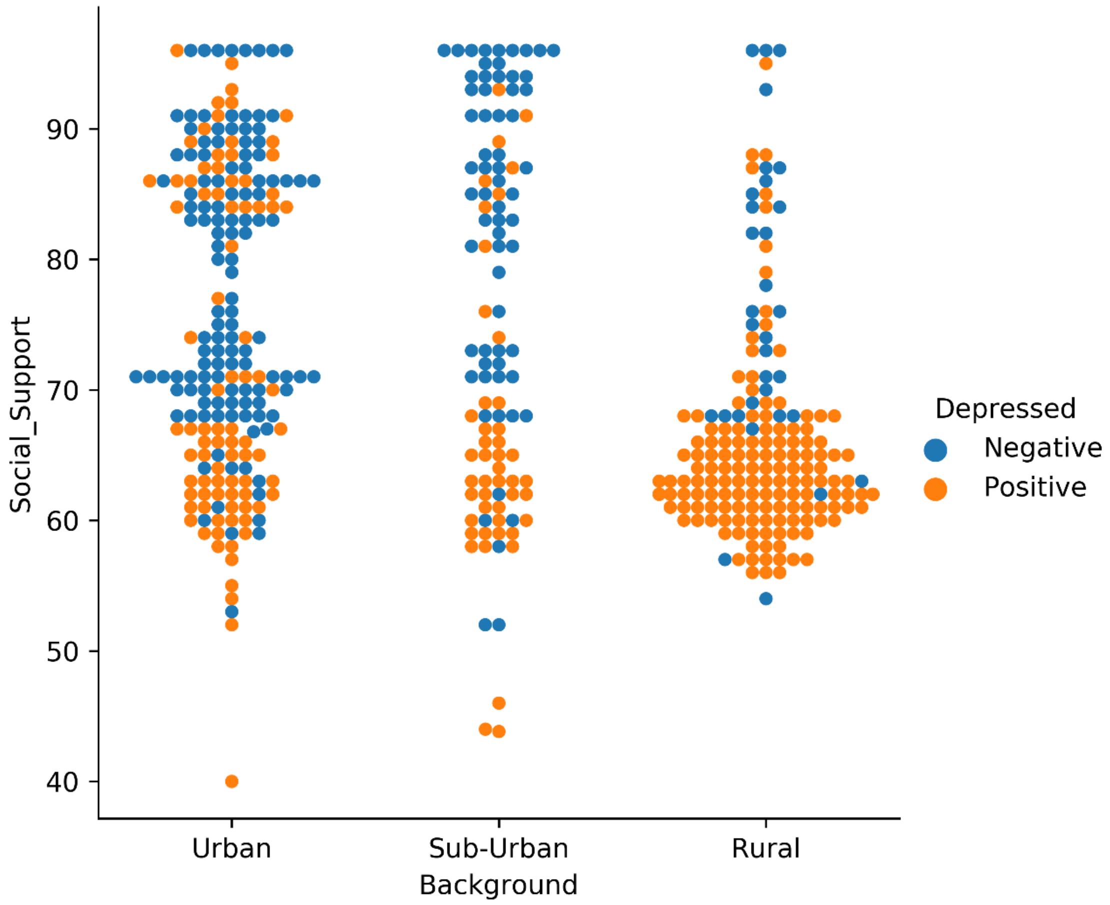

Figure S3 Association of social support and background of women with depression status

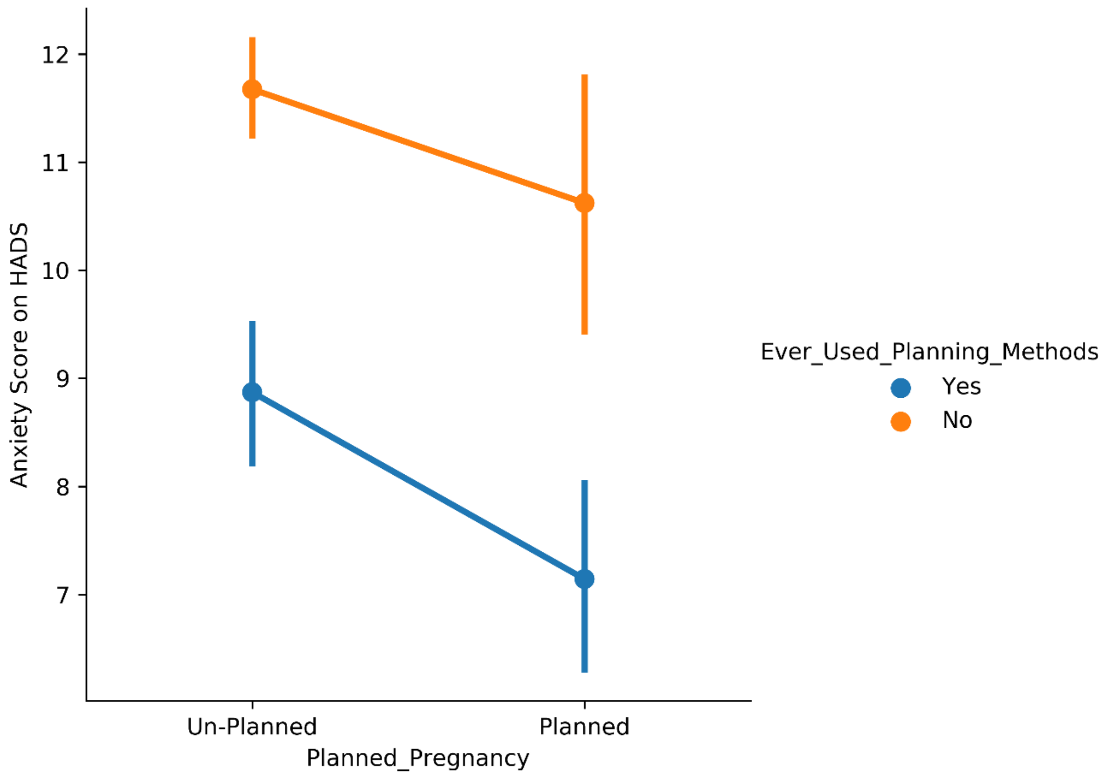

Figure S4 Use of reproductive planning and anxiety scores.

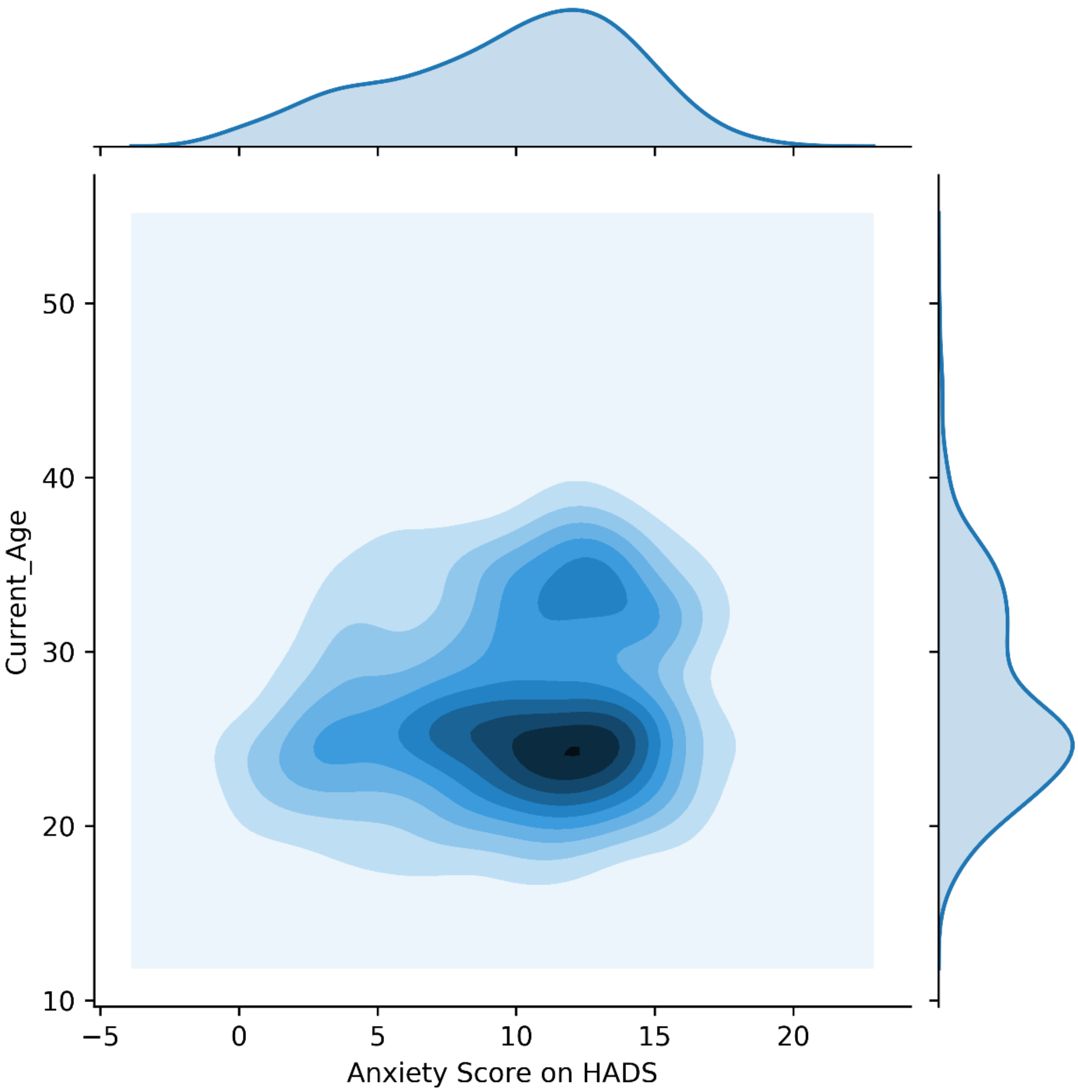

Figure S5 Age of pregnant women and scores of anxiety subscale.

Table S1: Demographic Characteristics of the Participants

Characteristics of participants	Category	(*f*)	(%)	Mean	Standard Deviation
Current Age				27.412	5.6532
Ethnicity	Punjabi	369	73.8%		
	Urdu	110	22.0%		
	Saraiki	10	<0.1%		
	Pashtun	7			
	Sindhi	2			
	Other	2			
Education	Illiterate	85	17%		
	Primary	83	16.6%		
	Middle School	103	20.6%		
	Matriculation	129	25.8%		
	Intermediate	60	12.0%		
	Graduate	31	6.2%		
	Masters	9	<0.1%		
Occupation	Housewife	441	88.2%		
	Employed	59	11.8%		
Duration Marriage				6.945	4.9121
Background	Rural	182	36.4%		
	Urban	208	41.6%		
	Sub-Urban	110	22.0%		
Maternal Age New				22.75	3.5584
Household Income	Low Income	148	29.6%		
	Lower Middle	284	56.8%		
	Upper Middle	58	11.6%		
	High Income	10	<0.1%		
Household Decision Maker	Self	21	<0.1		
	Husband	246	49.2%		
	In-laws	169	33.8%		
	Both	64	12.8%		
Fight/Arguments with In-laws	No	373	74.6%		
	Yes	127	25.4%		
Number of people living in the house	Less than or equal to 5	186	37.2%		
	More than 5	314	62.8%		
Smoking	No	490	>99.9%		
	Yes	10	<0.1%		

Substance Abuse	No	493	>99.9%		
	Yes	7	<0.1%		
Planned Pregnancy	Planned	135	27.0%		
	Un-Planned	365	73.0%		
Menstrual Cycle History	Regular	452	90.4%		
	Irregular	48	9.6%		
Ever Used Planning Methods	No	224	44.8%		
	Yes	276	55.2%		
Live Births				1.468	1.4146
Still Births				0.312	0.6659
Adverse Outcomes During Previous Pregnancy	No	413	82.6%		
	Yes	87	17.4%		
Abortion History	No	456	91.2%		
	Yes	44	8.8%		
Past Psychiatric Illnesses	No	492	>99.9%		
	Yes	8	<0.1%		
Psychiatric Illnesses in family	No	454	90.8%		
	Yes	46	9.2%		
Child Death	No	471	94.2%		
	Yes	29	5.8%		
Miscarriage	No	390	78.0%		
	Yes	110	22.0%		
Parents' Death	No	293	58.6%		
	Yes	207	41.4%		
Total Male Children				0.604	0.7876
Relationship Problems	No	465	93.0%		
	Yes	35	7.0%		
Long Illnesses	No	441	88.2%		
	Yes	59	11.8%		
Any other past trauma	No	486	>99.9%		
	Yes	14	<0.1%		
Harassment	No	467	93.4%		
	Yes	33	6.6%		
Ever Experienced Domestic Violence	No	491	>99.9%		
	Yes	9	<0.1%		

Total Spontaneous Vaginal Deliveries			0.836	1.2232
Total Episiotomies			0.232	0.6024
Total C-Section			0.436	0.8290
Total Female Children			0.902	1.0875
Social Support Questionnaire	Guidance		12.288	2.4093
	Nurturance		11.914	2.4598
	Reassurance		11.946	2.6903
	Reliable Alliance		12.478	2.3927
	Social Integration		11.846	2.7781
	Attachment		11.834	2.4303
	Social Support		72.315	12.201
